# Comparison of angioplasty, stents and conventional medical therapy for symptomatic intracranial atherosclerosis: a network meta-analysis

**DOI:** 10.3389/fneur.2026.1848611

**Published:** 2026-09-30

**Authors:** Jiawei Wang, Baogan He, Shoufeng Liu

**Affiliations:** 1 College of Clinical Medicine, China Medical University, Shenyang, China; 2 Department of Neurosurgery, Tianjin Huanhu Hospital, Tianjin, China

**Keywords:** angioplasty, drug-coated balloons, drug-eluting stents, network meta-analysis, stents, symptomatic intracranial atherosclerosis

## Abstract

**Background:**

Medical treatment and endovascular therapy are widely employed in the management of symptomatic intracranial atherosclerotic stenosis, however, the comparative effectiveness of these treatment modalities remains uncertain.

**Methods:**

We searched major Chinese and English databases to identify randomized controlled trials (RCTs) comparing four different endovascular modalities against other comparable non-endovascular therapies for patients with symptomatic intracranial atherosclerotic stenosis (sICAS). Eligible participants were individuals who had experienced a transient ischemic attack (TIA) or non-disabling stroke within the preceding 12 months. The literature search cutoff date was 11 January 2026.

**Results:**

For safety outcomes, the SUCRA ranking was as follows: medical treatment (MT) (SUCRA 90.1%) > drug-coated balloons (DCB) (SUCRA 52.8%) > balloon dilatation angioplasty (BDA) (SUCRA 40.7%) > bare metal stents (BMS) (SUCRA 35.4%) > drug-eluting stents (DES) (SUCRA 31.0%). MT exhibited a superior safety profile compared with BMS, with a statistically significant difference (RR = 0.47; 95% CI: 0.31 to 0.72). For long-term outcomes, exploratory SUCRA probability rankings were as follows: BDA (SUCRA 86.7%) > DES (SUCRA 77.4%) > MT (SUCRA 23.8%) > BMS (SUCRA 12.1%), and between-treatment differences were not statistically significant across all interventions.

**Conclusion:**

In terms of short-term safety, BMS treatment strategies were significantly inferior to medical treatment (MT), while no statistically significant differences in safety were observed among the various endovascular treatment modalities. In terms of adverse events during long-term follow-up, between-treatment differences were not statistically significant. Despite relatively high SUCRA values for BDA and DES for long-term outcomes, these rankings should be interpreted cautiously given the lack of statistically significant inter-group differences. Further research is required to verify the performance of each endovascular treatment strategy about restenosis.

**Systematic review registration:**

https://www.crd.york.ac.uk/PROSPERO/, CRD420251146704.

## 1 Introduction

Stroke is one of the leading causes of death and long-term disability worldwide (1). Intracranial atherosclerotic stenosis (ICAS) is an important cause of ischemic stroke and imposes a substantial disease burden in Asian populations (2). Symptomatic intracranial atherosclerotic stenosis (sICAS) generally refers to intracranial arterial stenosis that has triggered a recent transient ischemic attack or ischemic stroke, with the stenotic vessel confirmed as the culprit lesion. This condition may affect major intracranial arteries, including the intracranial internal carotid, middle cerebral, vertebral, and basilar arteries (3). The mechanisms underlying ischemic events associated with sICAS are complex, encompassing artery-to-artery embolism secondary to plaque rupture or thrombus formation, distal hypoperfusion, and progressive plaque growth. Compared with patients with mild or asymptomatic intracranial arterial stenosis, patients with sICAS who have recently experienced ischemic symptoms and have severe stenosis generally face a higher risk of recurrent stroke (4). Therefore, further reducing the risk of ischemic events in patients with high-risk sICAS remains an important clinical challenge in the prevention and management of cerebrovascular disease.

At present, medical therapy remains the cornerstone of secondary prevention in patients with sICAS. It generally comprises antiplatelet therapy, intensive lipid-lowering, and strict control of blood pressure and blood glucose. The American Academy of Neurology’s practice advisory emphasizes that patients with sICAS should receive antiplatelet therapy, intensive management of vascular risk factors, and lifestyle interventions as first-line treatment (3). Medical therapy reduces the risk of stroke primarily by inhibiting thrombus formation, stabilizing atherosclerotic plaques, and modifying systemic vascular risk factors; however, it cannot directly eliminate pre-existing arterial stenosis or mechanically improve the luminal diameter of the affected vessel. Patients with sICAS still face a measurable risk of recurrent stroke despite optimal medical therapy; among them, high-risk patients with severe stenosis and features of hemodynamic insufficiency, including hypoperfusion and inadequate collateral compensation, carry an even higher residual risk of ischemic events (5). Medical therapy alone may therefore be insufficient to adequately improve their hemodynamic status. Accordingly, endovascular intervention may be theoretically attractive for selected patients at high risk.

Endovascular treatment mainly includes strategies such as balloon angioplasty, drug-coated balloons, bare-metal stents, and drug-eluting stents (6). Balloon angioplasty can improve local blood flow by mechanically dilating the stenotic segment; however, its efficacy may be limited by elastic recoil, arterial dissection, acute vessel occlusion, and restenosis. Stent placement provides continuous radial support and may theoretically help maintain vessel patency, but it may also increase the risks of plaque displacement, distal embolization, in-stent thrombosis, and periprocedural stroke. The SAMMPRIS trial showed that, compared with aggressive medical therapy alone, Wingspan stenting combined with aggressive medical therapy significantly increased the risk of stroke or death within 30 days and did not demonstrate a clear clinical benefit during long-term follow-up (7). Subsequently, the CASSISS trial, despite employing strict patient selection criteria, delayed endovascular treatment, and rigorous operator qualification requirements, still failed to demonstrate that stenting plus medical therapy reduced the risk of stroke or death compared with medical therapy alone (8).

In recent years, clinical studies of balloon angioplasty and novel endovascular devices have increased steadily. The BASIS randomized clinical trial compared the efficacy and safety of balloon angioplasty combined with aggressive medical management versus aggressive medical management alone in patients with sICAS. This trial provided new randomized controlled evidence for evaluating the clinical value of balloon angioplasty in patients with sICAS (9). Furthermore, randomized controlled trials suggest that, compared with bare-metal stents, drug-eluting stents may reduce the risk of in-stent restenosis (10). Drug-coated balloons, by locally delivering antiproliferative agents while avoiding the implantation of a permanent device, may offer potential advantages in reducing restenosis and minimizing device-related risks (11, 12). However, existing studies differ to some extent in terms of patient selection, intervention protocols, and outcome measures. The relative efficacy and safety of different endovascular treatment strategies therefore remain insufficiently compared in a systematic manner.

Previous systematic reviews and meta-analyses have primarily focused on comparing stenting, balloon angioplasty, or endovascular treatment against best medical therapy. Although a previous network meta-analysis included strategies such as medical therapy, balloon angioplasty, and stenting (13), its literature search was conducted before the publication of several important randomized studies in recent years. Moreover, it did not adequately incorporate or distinguish newer interventional strategies, such as drug-coated balloons and drug-eluting stents. In addition, previous studies have generally grouped different endovascular techniques into a single treatment category and relatively few studies have directly compared balloon angioplasty, drug-coated balloons, bare-metal stents, and drug-eluting stents as separate treatment nodes. Therefore, the current evidence remains insufficient to clearly determine the relative efficacy and safety of different endovascular treatment strategies in patients with sICAS.

Network meta-analysis enables the simultaneous comparison of multiple interventions by integrating direct and indirect evidence, including comparisons for which direct head-to-head trials are unavailable. Therefore, we conducted a network meta-analysis comparing medical therapy, balloon angioplasty, drug-coated balloons, bare-metal stents, and drug-eluting stents as separate treatment nodes in patients with sICAS who had experienced TIA or non-disabling stroke within the prior 12 months. We evaluated the comparative efficacy and safety of these treatment strategies across clinically relevant outcomes, including recurrent ischemic stroke, any stroke, and all-cause mortality, and characterized their respective benefit–risk profiles. The findings may inform the selection of treatment strategies for patients with sICAS who have experienced TIA or non-disabling stroke, guide the design of future clinical trials, and support future updates of relevant clinical guidelines.

## 2 Methods

### 2.1 Protocol and registration

This study was prospectively registered on the PROSPERO database (Registration No. CRD420251146704). This study conformed to principles outlined in the Preferred Reporting Items for Systematic Reviews and Meta Analyses (PRISMA) extension statement for reporting systematic reviews incorporating network meta-analyses (14).

### 2.2 Eligibility criteria

#### 2.2.1 Participants

Eligible patients had a TIA or nondisabling stroke (modified Rankin Scale≤3) within 12 months before enrollment, attributed to angiographically verified stenosis of 70 to 99% of the diameter of a major intracranial artery.

#### 2.2.2 Interventions and comparisons

Studies comparing at least two of the following eligible interventions for symptomatic ICAS were included.

Angioplasty: Interventions included balloon dilatation angioplasty (BDA) or drug-coated balloon (DCB) therapy.

Stents: The interventions employed encompassed various types of stents, including bare metal stents (BMS), drug-eluting stents (DES).

Conventional medical therapy: This should be clearly defined and include at least single or dual antiplatelet agents, such as aspirin and clopidogrel, or statins.

#### 2.2.3 Outcomes

The present study must report at least one of the following quantitative outcome measures:

Primary safety outcome: Perioperative composite endpoint events including any stroke (ischemic or hemorrhagic) or all-cause mortality. A uniform 30-day post-procedure time window was applied for the safety outcome in this study, and data from included trials with comparable time windows were extracted per this standard.

Primary efficacy outcome: Adverse events including any stroke, all-cause death, and TIA occurring during long-term follow-up (including the initial 30-day post-procedural period).

#### 2.2.4 Study types

We included randomized controlled trials (RCTs). Other types of studies including case series and case reports were excluded.

#### 2.2.5 Search

We searched major Chinese and English databases, including EMBASE, Web of Science (WOS), PubMed, Cochrane Library, Chinese Biomedical Literature Database (CBM), China National Knowledge Infrastructure (CNKI), VIP Chinese Journal Database (VIP), and WanFang Data. The following keywords were used and limited to the title and abstract: (“Intracranial Arteriosclerosis” or “Cerebral Atherosclerosis”) and (“stents” or “angioplasty”) and (“Randomized Controlled Trial” or “Random”). Search strategy was drafted according to the Peer Review of Electronic Search Strategies checklist, and the details of the strategy are provided in Supplementary Tables 1–8.

### 2.3 Data

#### 2.3.1 Data collection

Data were independently extracted by two reviewers and transcribed into a dedicated database. In cases of discrepancies, a third reviewer was consulted. The data extracted from each report included the first author, publication year, study type, sample size, patient characteristics, study and control treatments, follow-up duration, and outcome data.

#### 2.3.2 Risk of bias

Risk of bias assessment was conducted by 2 authors, Cochrane Risk of Bias 2.0 (RoB 2) instrument was employed to independently evaluate the methodological quality and risk of bias of each identified RCT (15). Publication bias was evaluated by Egger’s test and visualized using funnel plots.

#### 2.3.3 Data synthesis and statistical analysis

Network meta-analysis was performed using STATA software (Version 16.0) with the mvmeta command under a frequentist fixed-effect consistency-assumption model (16). Given that we applied a fixed-effect model, the between-study heterogeneity variance (τ2) was not estimated, as this parameter is not incorporated within the fixed-effect modeling framework. We performed descriptive statistical analyses and calculated the I^2^ statistic for each paired comparison to assess the presence of clinical and methodological heterogeneity both within and across treatment comparisons. For studies containing zero-event cells in 2 × 2 contingency tables, the Haldane-Anscombe 0.5 continuity correction was applied, whereby 0.5 was added to all four cells of the contingency table to avoid computational failure when calculating relative risks (RRs). The relative effects of different treatment modalities were evaluated using Relative Risks(RRs), 95% confidence intervals (95% CIs), and predictive intervals (PrIs). Forest plots, league tables, and SUCRA probability rankings were also utilized for the presentation of statistical results (17).

## 3 Results

### 3.1 Study selection

A total of 1710 articles were retrieved from the databases through systematic searching. To ensure the inclusion of the most relevant and methodologically rigorous studies, a meticulous screening process was implemented, as illustrated in the PRISMA flow diagram (Figure 1). After excluding 236 duplicate articles, the remaining 1,474 articles underwent title and abstract screening, with 12 articles ultimately included (7–11, 18–24). Among these, two were different follow-up reports of the same RCT (7, 18). When more than one report of the same trial was available, only the report with the longest follow-up or the outcome data matching the prespecified time window was used, and patients were not counted more than once. Since the outcome indicators reported varied across articles, only those documenting the required outcome indicators were included for the meta-analysis of each specific outcome. Table 1 and Supplementary Table 14 summarize the characteristics of each included article, including sample size, PICOS elements, and follow-up duration.

**Figure 1:**
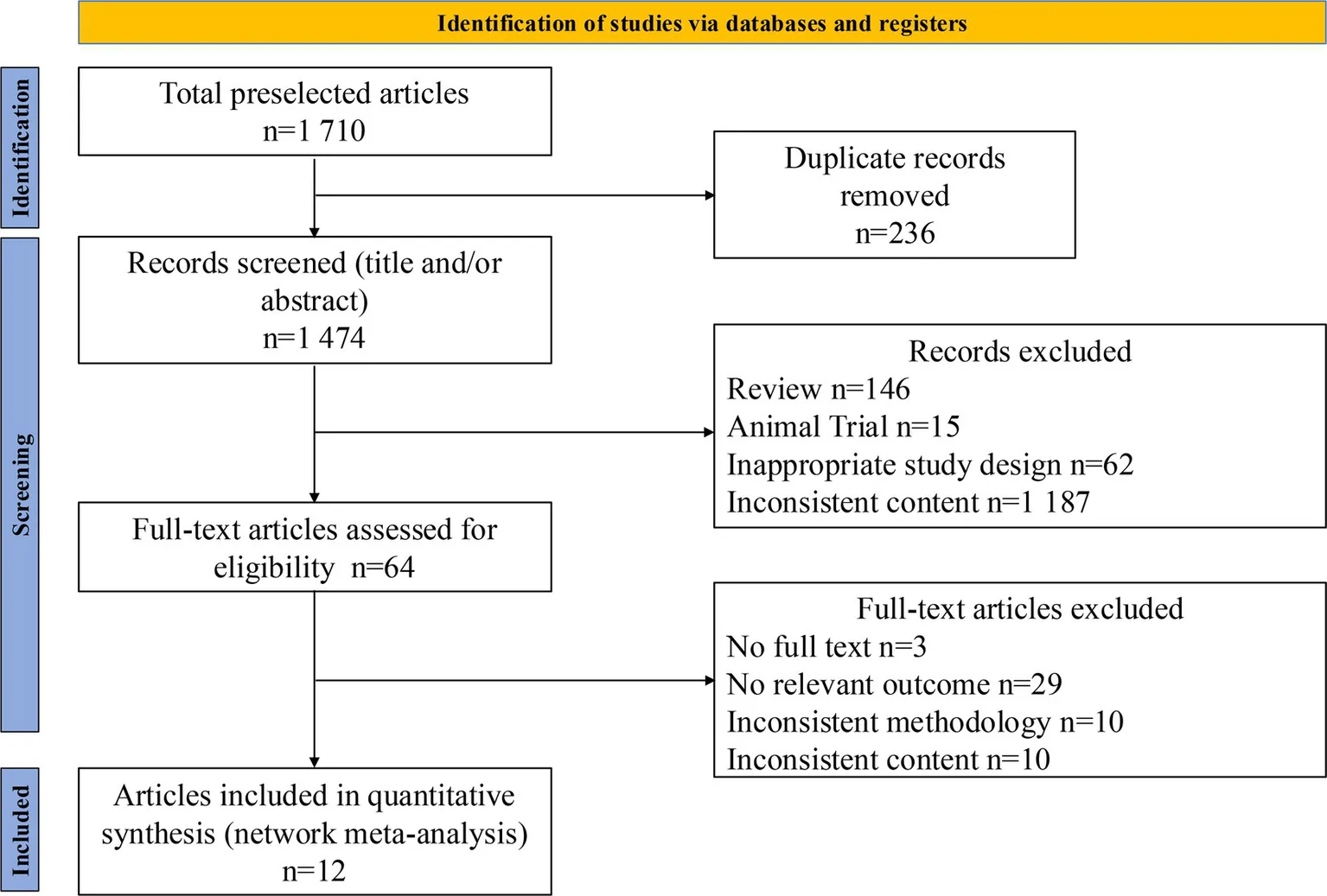
PRISMA flow diagram.

**Table 1:** Baseline characteristics of the included studies.

Study	Participants, *n*	Treatment	Age, y	Degree of stenosis, %	Outcomes
Sun et al. (9)	249	BDA	58	80.2355	①②
	252	MT	58	79.1975	
Ma et al. (11)	90	DCB	59	84.5	①③
	90	BMS	59	80	
Jia et al. (10)	132	DES	59	76.8	①②③
	131	BMS	58	74.8	
Gao et al. (8)	176	BMS	56.7	78.5	①②
	182	MT	55.9	76.6	
Ip et al. (21)	74	BMS	62	78	①②
	76	MT	61	78	
Chimowitz et al. (18)	224	BMS	61	80	①
	227	MT	59.5	81	
Xiao et al. (23)	48	BMS	65.2	81.9	①
	44	MT	66.4	82.1	
Huang et al. (20)	71	BMS	61.09	NR	②
	71	MT	60.28	NR	
Zaidat et al. (24)	58	BMS	61.8	78.9	①②
	53	MT	61.8	80.4	
Si et al. (22)	92	DES	61.7	82.7	①②③
	96	BMS	61.5	84.3	
He et al. (19)	20	DES	58.6	80	①②③
	20	BMS	61.6	82.5	

① indicates Stroke or Death within 30 days; ② indicates Stroke or Death within 1 year; ③ indicates restenosis rate. NR = not reported.

### 3.2 Assessment of risk of bias, heterogeneity, transitivity, and statistical inconsistency

The risk of bias for each included study is provided in the Supplementary Table 9. Among the 11 studies, 8 (72.7%) exhibited a low risk of random-sequence generation bias. All studies were assessed as having a low risk of deviation from intended interventions. Nine studies (81.8%) had a low risk of missing outcome data, 10 studies (90.9%) showed a low risk of measurement of the outcome domain, and Selection reporting bias was not found in 11 studies. Overall, 3 studies (27.3%) were categorized as having a high risk of bias (Figure 2). No heterogeneity was detected for safety outcomes, whereas substantial within-group and between-group heterogeneity was identified for long-term outcomes (Figure 3). Variations in the completeness of baseline data and differences in background medical treatment regimens across studies may represent one potential source of heterogeneity in long-term outcomes. Regarding transitivity, we performed quantitative analyses of patient age, baseline stenosis severity, baseline modified Rankin Scale (mRS) score, and time from the qualifying event to intervention (Figure 4). We also conducted qualitative analyses of lesion location and background medication for included participants. Based on the combined findings from these analyses, all evidence networks adhered to the principle of transitivity. The Egger’s test was employed to assess publication bias, and funnel plot analyses revealed no or minimal publication bias (Figure 5). However, given the limited number of included studies, these analyses could not reliably rule out publication bias.

**Figure 2:**
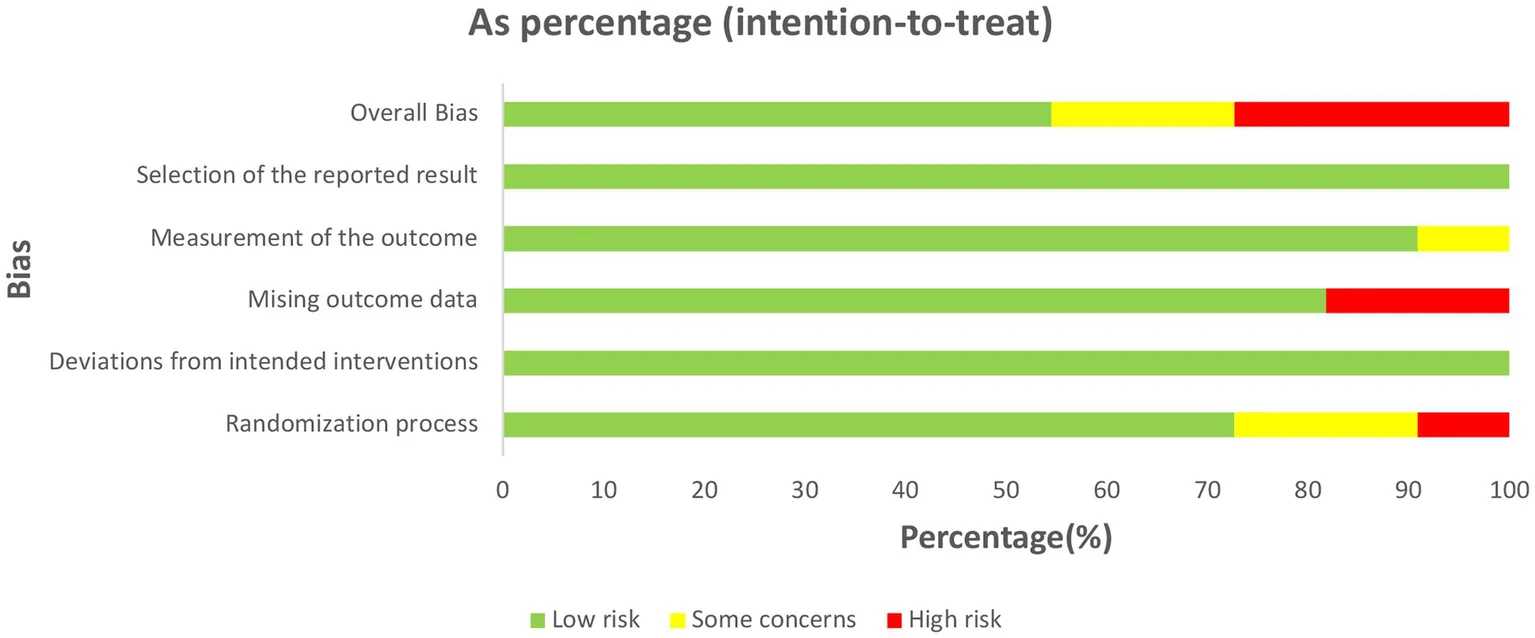
Overall risk of bias presented as percentage of each risk of bias item across all included studies. Green = Low risk, Red = High risk, Yellow = Some concerns.

**Figure 3:**
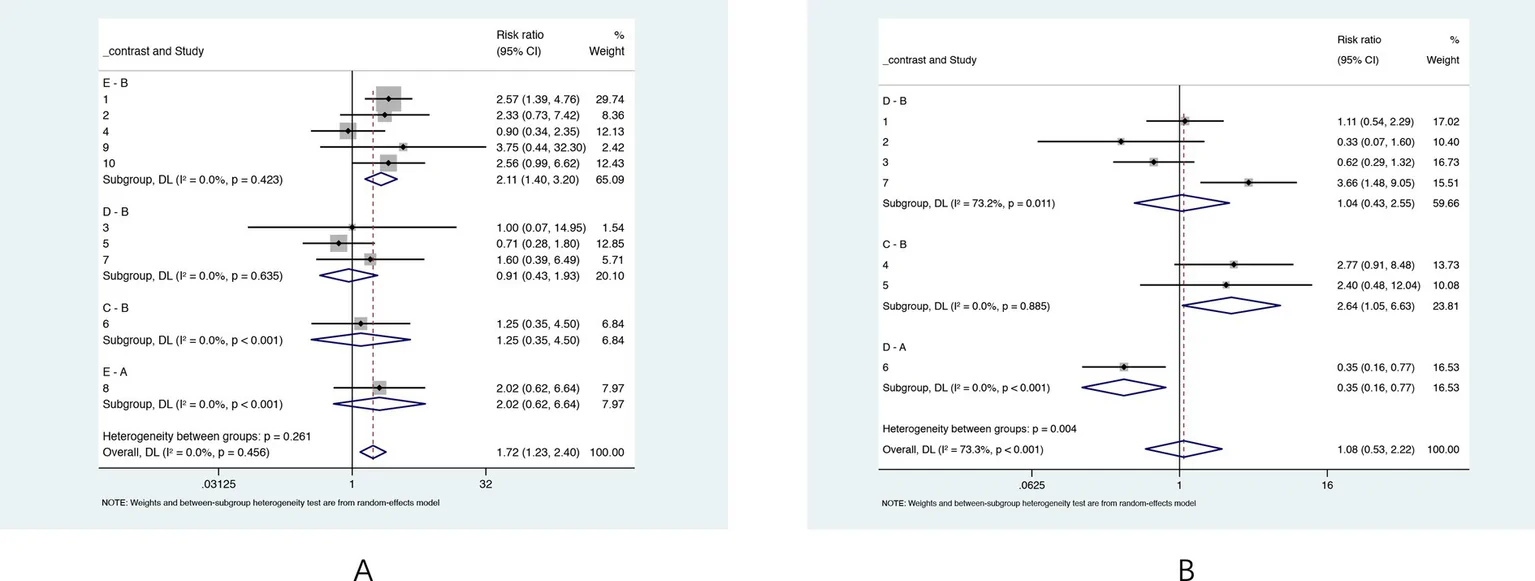
Heterogeneity analyses for all outcomes including stroke or death within 30 days **(A)** and stroke and death over the course of long-term follow-up **(B)**.

**Figure 4:**
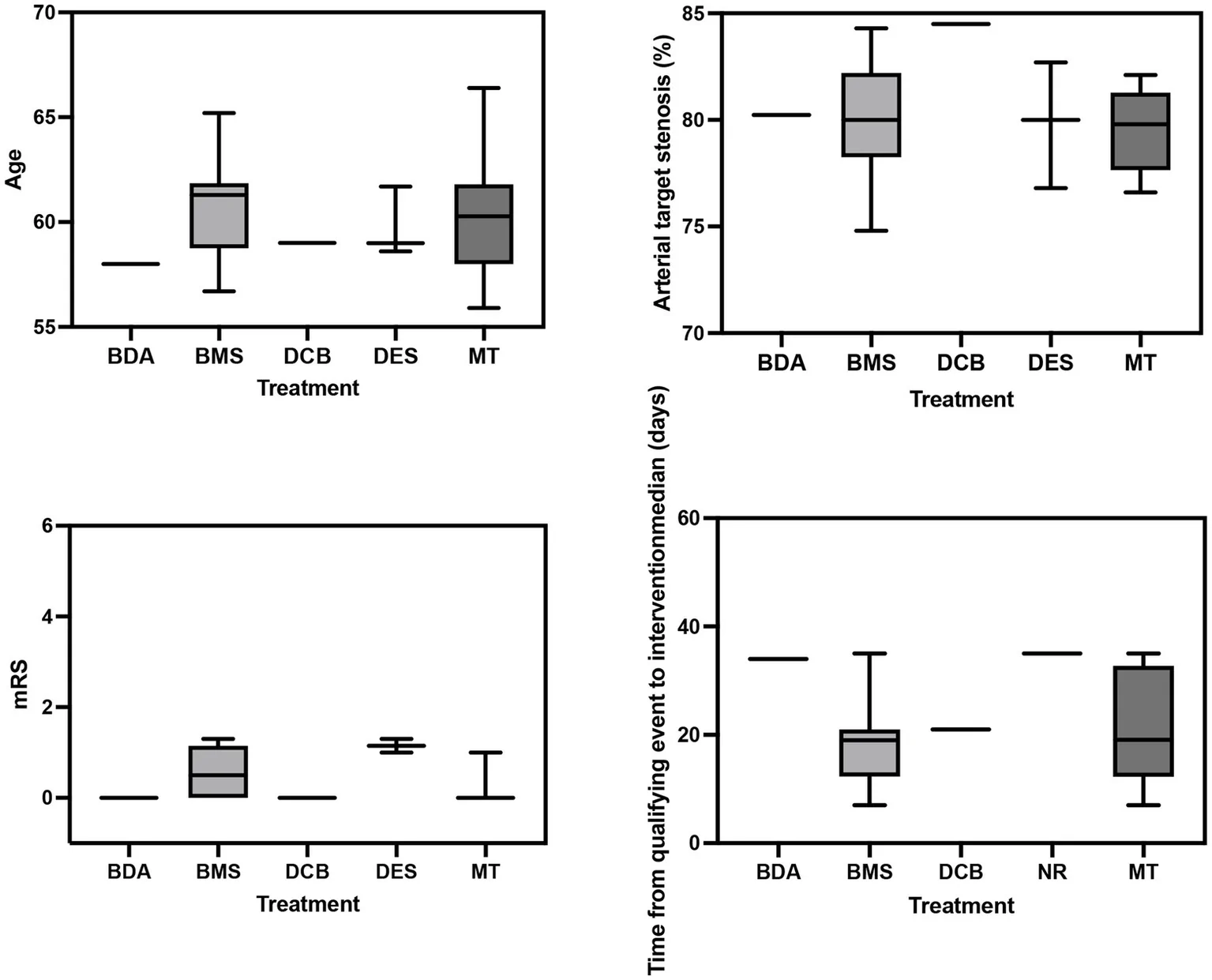
Transitivity assessment.

**Figure 5:**
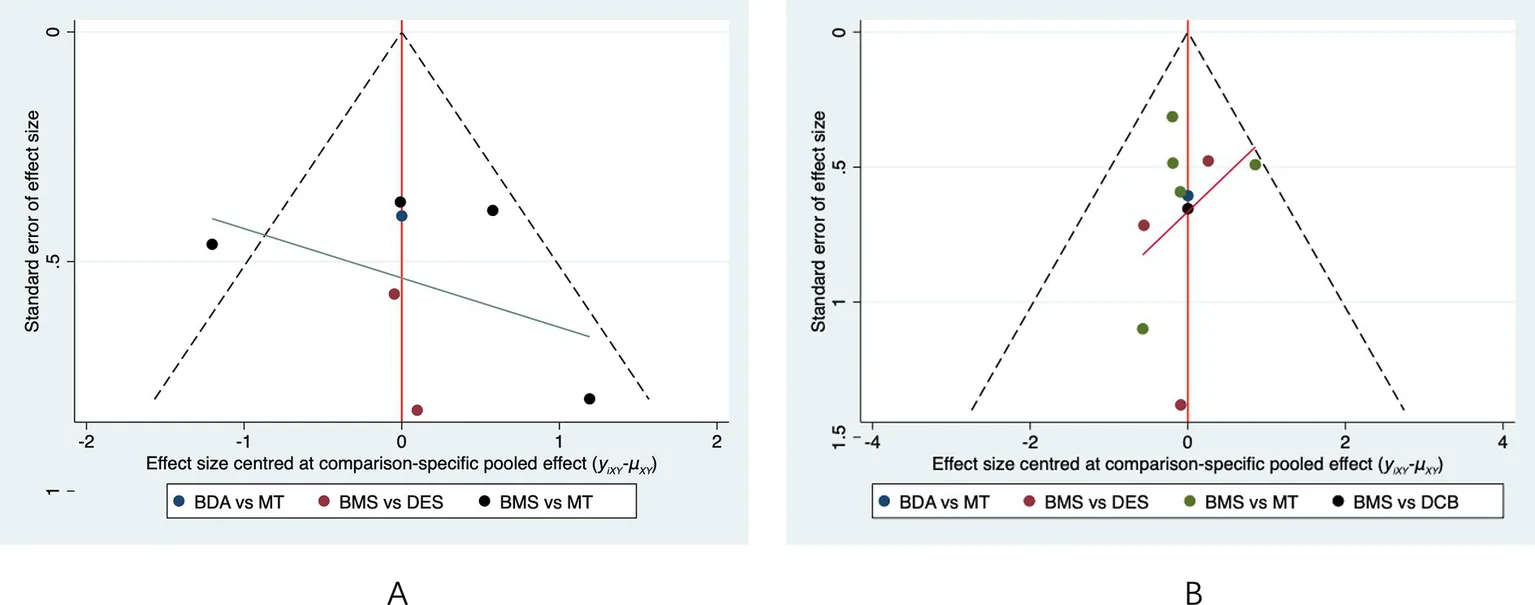
Funnel plot of all outcomes including stroke or death within 30 days **(A)** and stroke and death over the course of long-term follow-up **(B)**.

### 3.3 Safety outcomes

For 30-day mortality and stroke, the network meta-analysis included 10 studies involving 2,334 participants. The included studies encompassed five intervention modalities, namely bare-metal stent (BMS), drug-coated balloon (DCB), drug-eluting stent (DES), balloon dilatation angioplasty (BDA), and medical treatment (MT). As indicated by the evidence network plot, the bare-metal stent (BMS) group had the largest sample size, which was represented by the largest node in the plot. Additionally, the largest number of studies compared BMS with conventional medical treatment, corresponding to the thickest line connecting the two nodes in the plot. Furthermore, due to the paucity of relevant randomized controlled trials (RCTs), the network failed to form a closed loop (Figure 6). Closed loops are a prerequisite for loop-specific inconsistency testing, which quantitatively evaluates the agreement between direct and indirect evidence for a given treatment comparison. Since no closed loops were present in the safety-outcome network, formal statistical inconsistency assessment could not be implemented. All comparative effect estimates from this network meta-analysis are therefore built upon the untested consistency assumption. Potential discrepancies between direct and indirect evidence cannot be statistically ruled out, which brings extra uncertainty to our treatment-effect estimates.

**Figure 6:**
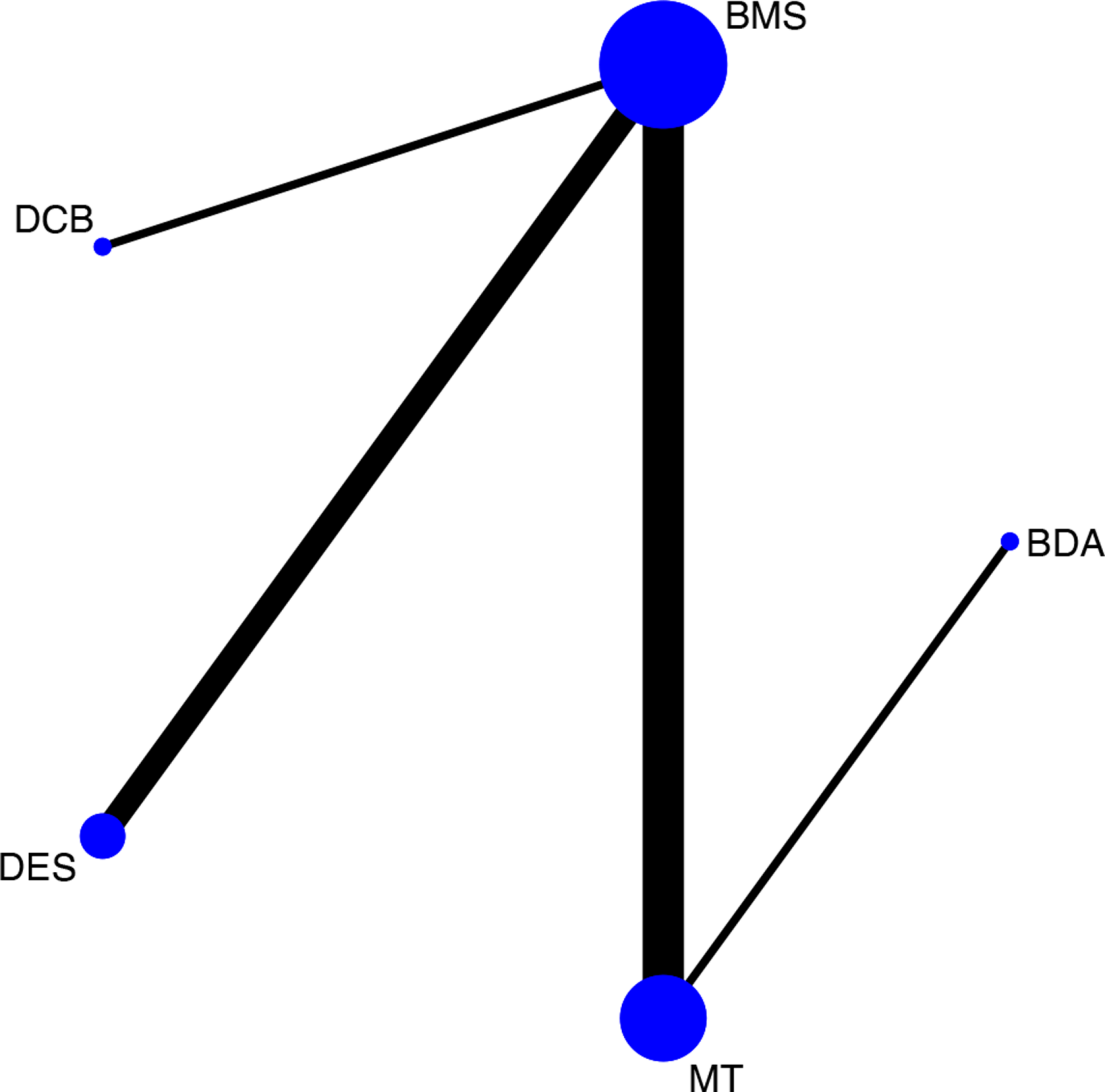
Network plot for different treatment in safety outcomes.

The forest plot demonstrated that MT exhibited a superior safety profile compared with BMS, with a statistically significant difference (RR = 0.47; 95% CI: 0.31 to 0.72). No statistically significant differences were observed among the remaining treatment strategies (Figure 7). For all relative-risk comparisons presented in this forest plot, RR < 1 favors the first-listed treatment in each pairwise comparison. The SUCRA ranking was as follows: MT (SUCRA 90.1%) > DCB (SUCRA 52.8%) > BDA (SUCRA 40.7%) > BMS (SUCRA 35.4%) > DES (SUCRA 31.0%) (Figure 8; Table 2; Supplementary Figure 1; Supplementary Table 10).

**Figure 7:**
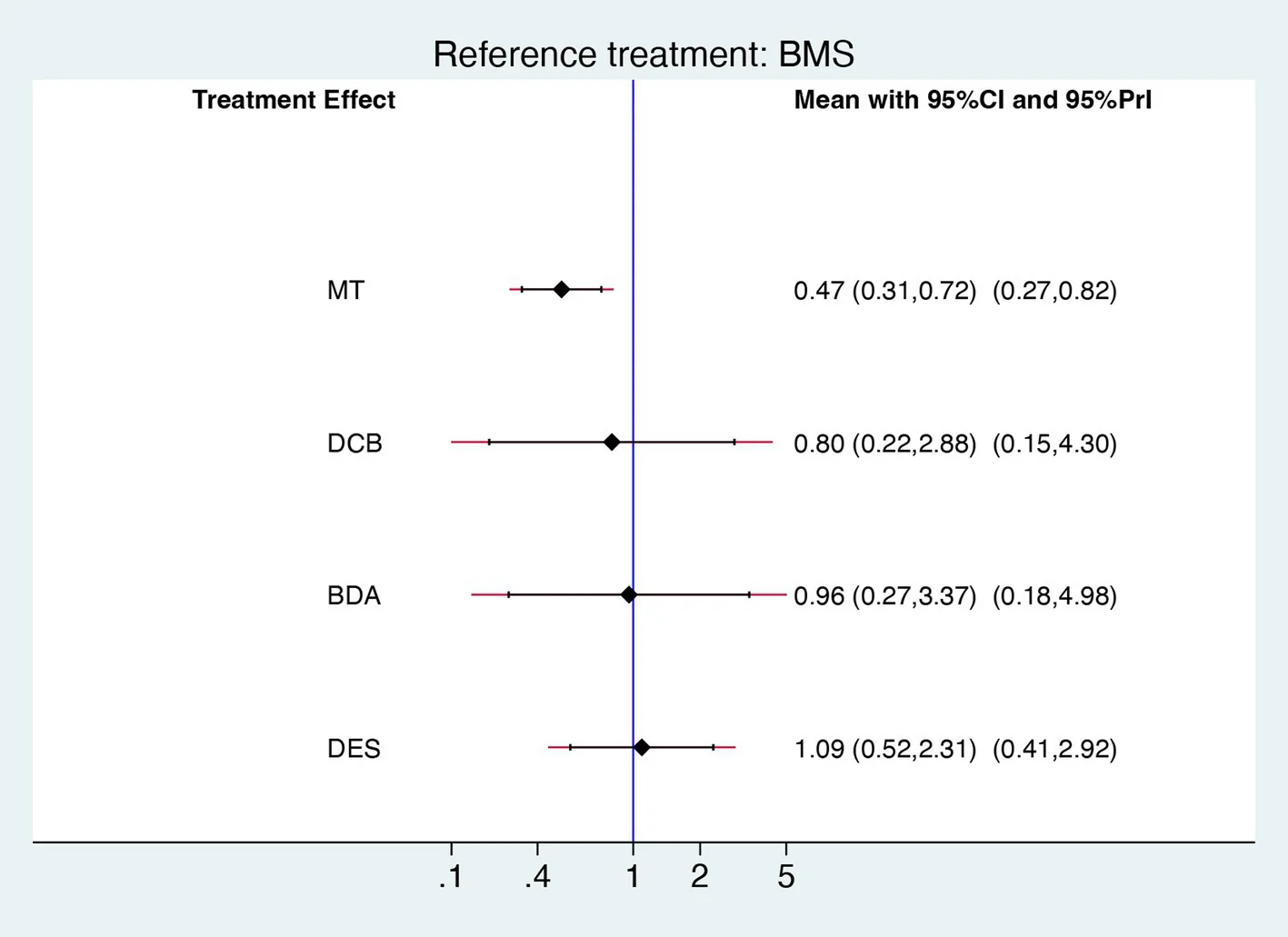
Forest plots for different treatment in safety outcomes.

**Figure 8:**
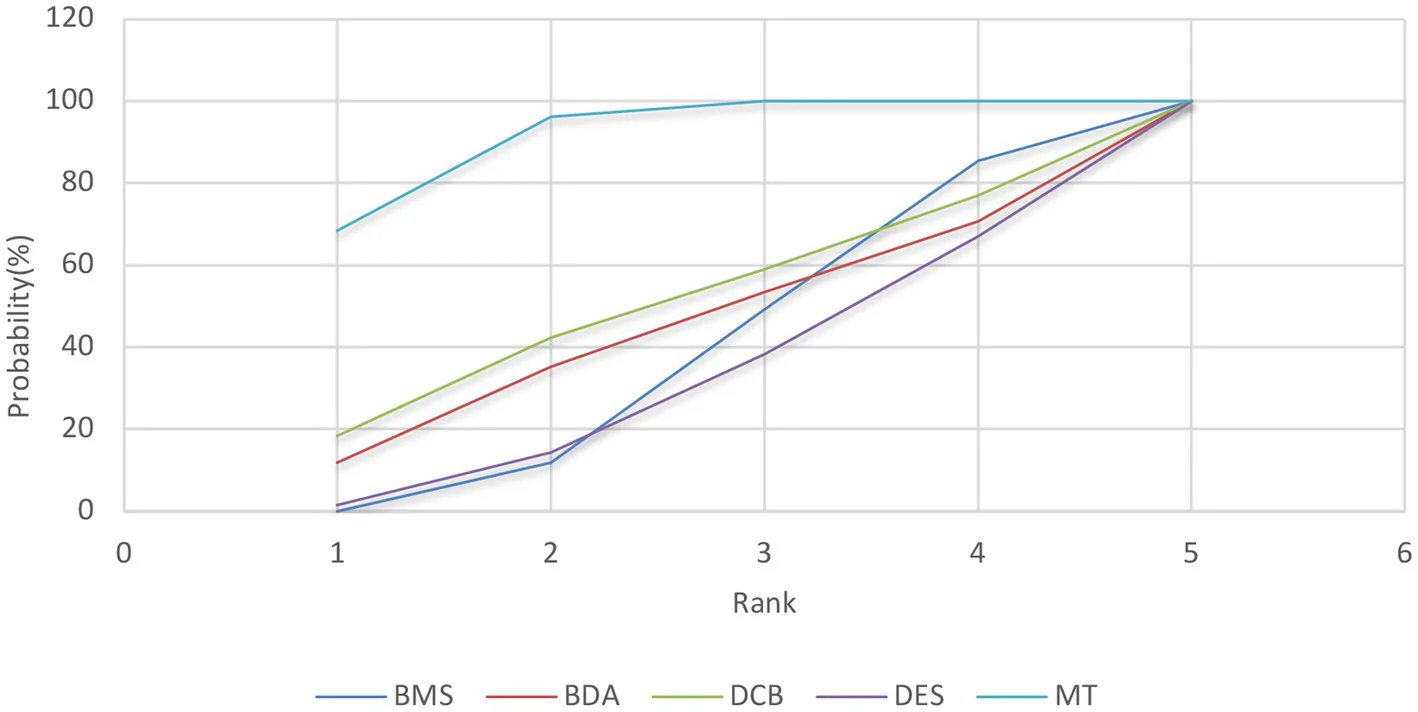
Ranking plots for different treatment in safety outcomes.

**Table 2:** SUCRA of the different treatment in safety outcomes.

Treatment	SUCRA	PrBest	Mean rank
MT	90.1	66.1	1.4
BDA	40.7	10	3.4
BMS	35.4	0	3.6
DCB	52.8	21.7	2.9
DES	31	2.2	3.8

### 3.4 Any stroke, death or TIA during the long‑term follow‑up

This analysis included 8 studies, encompassing 1712 patients with outcomes of stroke, death or TIA within one year. The evidence network indicated that there were a greater number of studies comparing bare metal stents (BMS) with medical treatment (MT) (Figure 9). Similarly, the evidence network for long-term follow-up outcomes also lacked closed loops; accordingly, formal inconsistency testing was not feasible for this endpoint.

**Figure 9:**
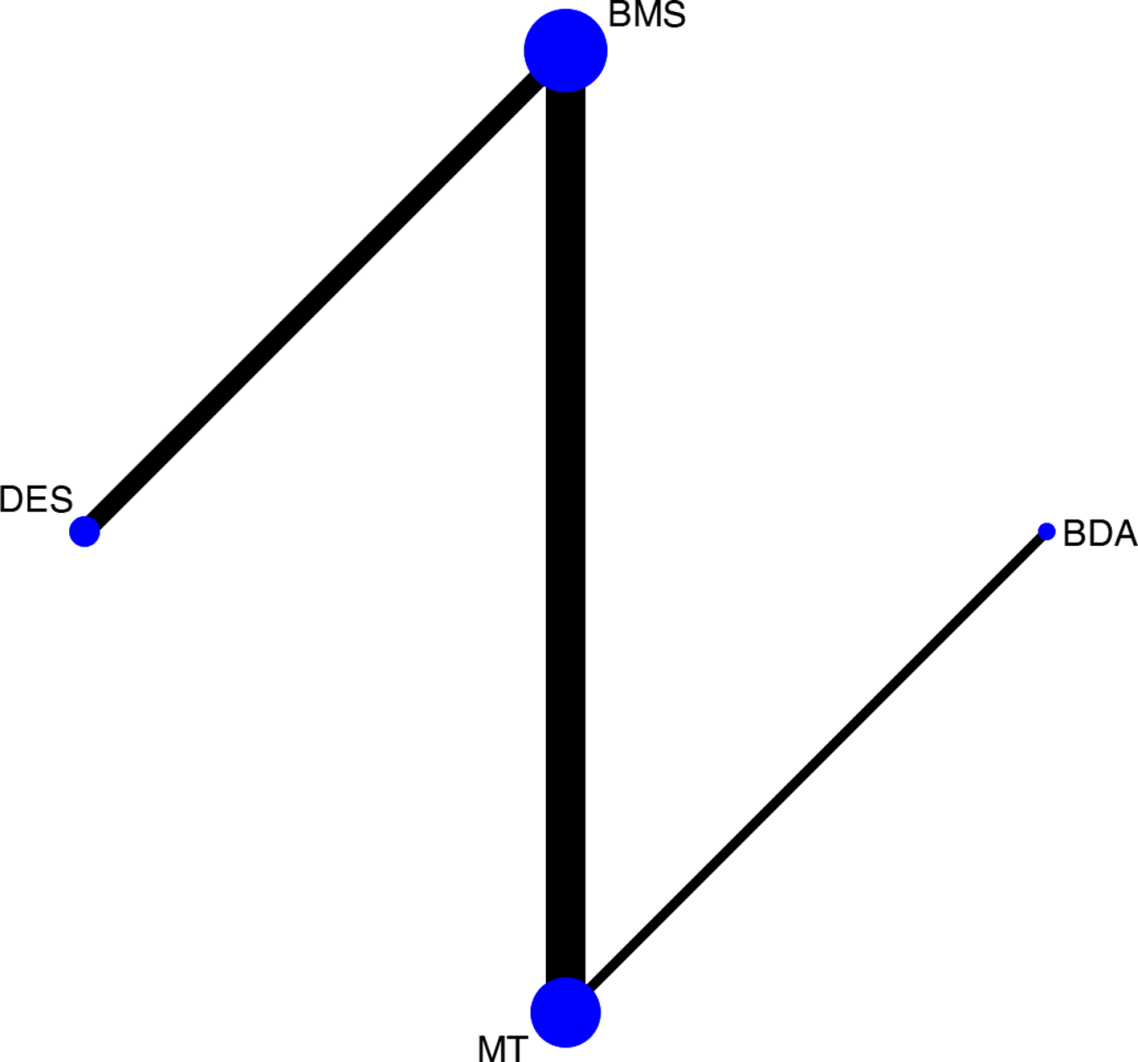
Network plot for different treatment in any stroke or death during the long-term follow-up.

Forest plots showed that between-treatment differences were not statistically significant for long-term adverse event outcomes across all included interventions (Figure 10). RR < 1 favors the first-listed treatment for pairwise comparisons in this forest plot. Exploratory SUCRA probability rankings for long-term outcomes were as follows: BDA (SUCRA 86.7%) > DES (SUCRA 77.4%) > MT (SUCRA 23.8%) > BMS (SUCRA 12.1%) (Figure 11; Table 3; Supplementary Figure 2; Supplementary Table 11). These SUCRA values represent ranking probabilities only and do not indicate proven clinical superiority of BDA or DES.

**Figure 10:**
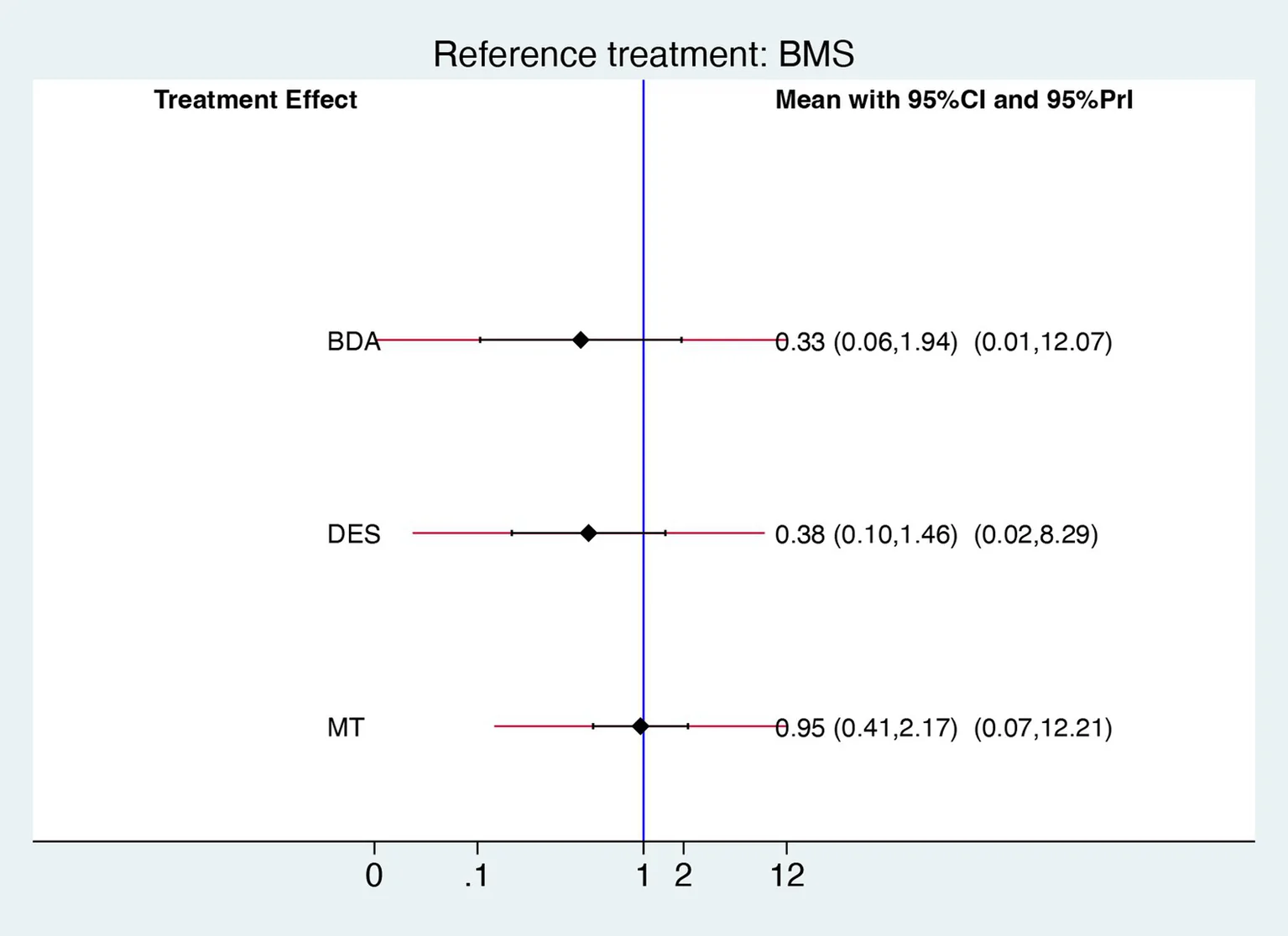
Forest plots for different treatment in any stroke or death during the long-term follow-up.

**Figure 11:**
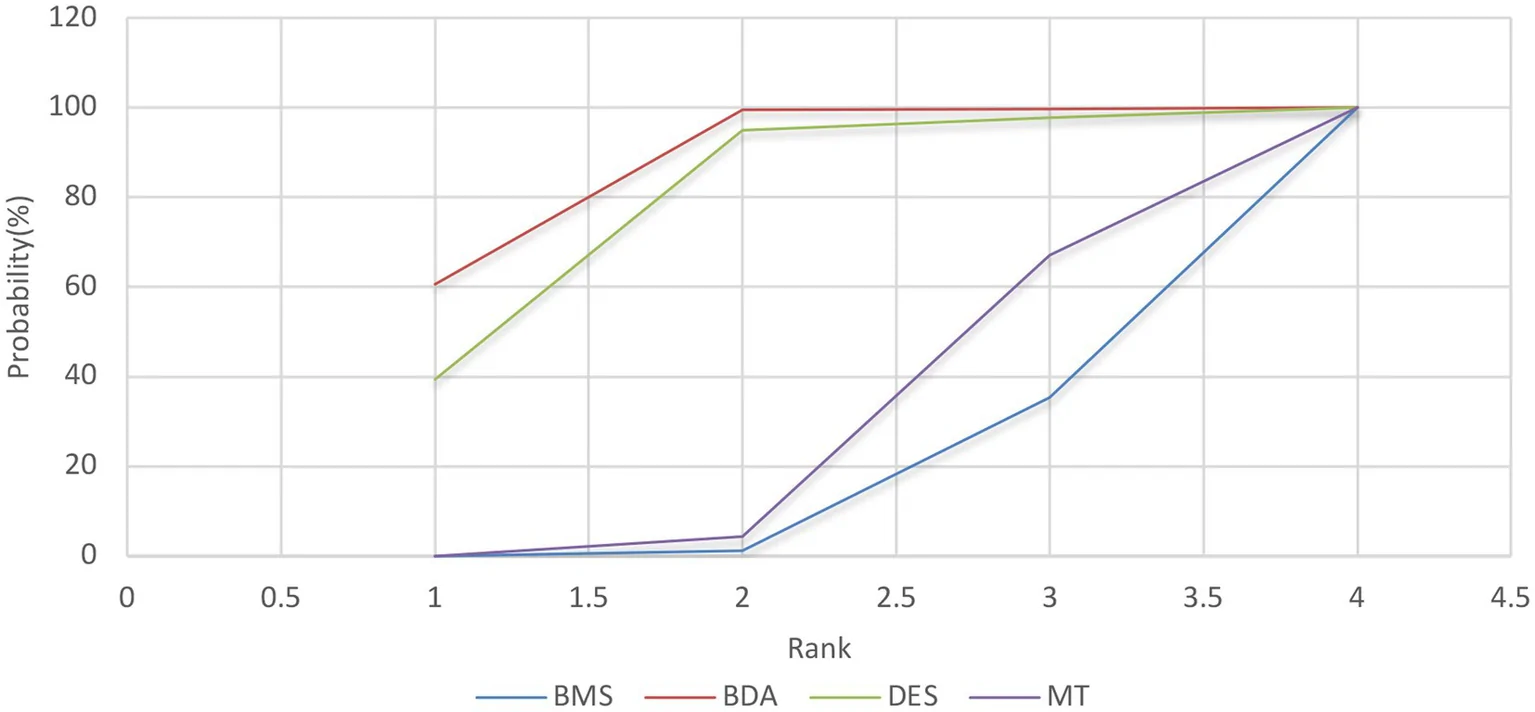
Ranking plots for different treatment in any stroke or death during the long-term follow-up.

**Table 3:** SUCRA of different treatment in any stroke or death during the long-term follow-up.

Treatment	SUCRA	PrBest	Mean rank
MT	23.8	0	3.3
BDA	86.7	60.6	1.4
BMS	12.1	0	3.6
DES	77.4	39.4	1.7

### 3.5 Sensitivity analyses and certainty of evidence

To further examine the sensitivity of the findings, we performed a sensitivity analysis excluding two trials (He et al., 2019; Si et al., 2022) with population-mismatch concerns. These sensitivity analyses proved consistent with the primary results, confirming the robustness of our findings (Table 4). After assessing the level of evidence using the CINeMA framework, most pairwise comparison results for safety outcomes demonstrated moderate confidence, while those for long-term adverse events showed low (Supplementary Figures 3–6; Supplementary Tables 12, 13) (25). This may be attributed to the relatively small number of included studies.

**Table 4:** Sensitivity analyses.

Treatment	Stroke or Death WITHIN 30 days		Stroke or death during the long term follow up	
	Main estimate	Sensitivity estimate	Main estimate	Sensitivity estimate
BDA	0.96 (0.27, 3.37)	0.96 (0.27, 3.37)	0.33 (0.06, 1.94)	0.47 (0.16, 1.35)
DCB	0.80 (0.22, 2.88)	0.80 (0.22, 2.88)	NA	NA
DES	1.09 (0.52, 2.31)	1.42 (0.56, 3.61)	0.38 (0.10, 1.46)	0.36 (0.11, 1.18)
MT	0.47 (0.31, 0.72)	0.49 (0.31, 0.79)	0.95 (0.41, 2.17)	1.34 (0.74, 2.42)

## 4 Discussion

### 4.1 Principal findings

This network meta-analysis comprehensively evaluated and compared the efficacy and safety of various endovascular interventions as well as conventional medical treatment for symptomatic intracranial arterial stenosis, including the latest DCB and DES technologies. Based on data from 11 eligible RCTs involving 2,476 participants, we assessed the incidence of stroke and mortality within 30 days, as well as the incidence of adverse events during long-term follow-up. MT demonstrated the highest cumulative ranking probability for safety outcomes (SUCRA, 90.1%) and was significantly safer than BMS (RR, 0.47; 95% CI, 0.31–0.72). For long-term outcomes, BDA and DES ranked highest according to SUCRA, with values of 86.7 and 77.4%, respectively; however, no statistically significant differences were observed among the treatment strategies.

### 4.2 Comparisons

#### 4.2.1 Comparison of safety outcomes

In the present study, MT ranked highest with respect to safety outcomes and demonstrated a statistically significant safety advantage over BMS. The risk of safety-related events for MT was approximately 47% of that observed with BMS, translating to a relative risk reduction of around 53%. This finding may be explained by the fact that MT obviates catheter manipulation, balloon angioplasty, or stent implantation, thereby averting intervention-related complications such as vascular dissection, vascular perforation, plaque shift, perforator artery occlusion, and technical failure. In comparison, besides inherent periprocedural operative risks, BMS may impose additional risks of in-stent thrombosis, neointimal hyperplasia, restenosis, and bleeding linked to long-term antiplatelet therapy (26, 27).

Between-group differences failed to attain statistical significance. Nevertheless, DCB ranked second in the SUCRA-derived hierarchy (SUCRA = 52.8%), indicating a potential relative safety advantage. DES obtained the lowest SUCRA rank (SUCRA = 31.0%). Although local delivery of antiproliferative agents by DES suppresses neointimal hyperplasia and mitigates in-stent restenosis risk, its implantation procedure, delayed endothelialization, stent-related thrombosis and long-term antiplatelet-associated hazards may limit safety benefits (28, 29).

#### 4.2.2 Comparison of long-term outcomes

In contrast to safety-related outcomes, BDA and DES achieved relatively high SUCRA-based rankings for long-term efficacy outcomes, at 86.7 and 77.4%, respectively, whereas MT and BMS obtained lower ranks. This suggests that certain endovascular treatment strategies may confer relative advantages with respect to long-term efficacy. From a mechanistic perspective, BDA dilates stenotic vessels, improves local blood flow and cerebral tissue perfusion, and alleviates hemodynamic impairment. It may also exert a partial protective effect against long-term ischaemic events attributable to hypoperfusion (30). Through sustained release of antiproliferative agents, DES inhibits neointimal hyperplasia and lowers the risk of restenosis, contributing to the preservation of relatively stable vascular patency over long-term follow-up (31).

Nevertheless, between-treatment differences were not statistically significant for long-term outcomes, which may be attributable to the limited number of included studies and insufficient sample sizes. Furthermore, long-term outcomes are not solely determined by devices or treatment modalities per se; they can be jointly modulated by multiple factors, including patients’ baseline risk profiles, treatment adherence, anatomical characteristics of lesions, ischemic mechanisms, collateral-circulation compensation status, as well as periprocedural management and the quality of risk-factor control. This multifactorial background may partly account for the substantial within-group and between-group heterogeneity observed for long-term outcomes in our analysis. The included RCTs differed with respect to patient-level characteristics, such as lesion morphology, collateral circulation status and overall vascular risk burden. Trial intervention protocols were also inconsistent, including variations in endovascular procedural techniques and the intensity of background medical therapy. Despite our prespecified preference for outcome data with ≥12-month follow-up, the actual follow-up windows still varied across studies; a small number of trials reported events at 6-month follow-up, which could further contribute to the observed heterogeneity. Accordingly, a high SUCRA rank for a given treatment strategy cannot be simply attributed to the intrinsic performance advantages of the device itself. Future high-quality randomized controlled trials with adequate sample sizes and prolonged follow-up periods are warranted to further validate the genuine long-term clinical benefits of BDA and DES across diverse patient subgroups.

### 4.3 Clinical implications

The present study suggests that clinical values of different therapeutic strategies exhibit temporal-dimension differences for patients with sICAS who have experienced TIA or non-disabling stroke, and a trade-off should be made between safety and long-term benefits. Medical treatment can obviate early-phase risks originating from endovascular manipulation and yields the optimal safety profile; hence, it should serve as the foundational therapeutic strategy, particularly for patients with high periprocedural risks and no definite hemodynamic impairment. Balloon angioplasty and drug-eluting stents show more favorable long-term outcomes, characterized by the profile of “early procedural risks accompanied by potential long-term benefits”. It should be highlighted that we applied the CINeMA framework to assess the certainty of evidence. Unlike most comparisons for safety outcomes that yielded moderate certainty, the majority of pairwise comparisons for long-term adverse events were rated as low or very low certainty. This insufficient certainty of evidence should be fully taken into account when extrapolating our long-term findings to clinical practice. For patients who suffer from recurrent ischemic events despite adequate standardized medical therapy, together with high-risk features such as confirmed hypoperfusion and poor collateral circulation, endovascular therapy could be considered as an individualized alternative. Nevertheless, its implementation demands strict patient selection and thorough pre-procedural assessment, with full consideration of multiple factors including lesion location, stenosis severity, plaque characteristics, collateral status, and operator experience. Furthermore, Asian participants accounted for 82.5% (1928/2337) of the pooled population in this study. Accordingly, our findings carry certain implications for Asian regions with a high prevalence of symptomatic intracranial atherosclerosis, and may act as an important evidence base for the management of this disease in Asian populations. Finally, we acknowledge that our study exclusively enrolled patients with sICAS presenting with TIA and non-disabling stroke. In the setting of acute stroke, more appropriate treatment strategies including mechanical thrombectomy and intravenous thrombolysis are available. As such, the findings of the present study have limited applicability to stroke events in the acute setting.

### 4.4 Limitations

The present study possesses several inherent limitations. First, relatively few randomized controlled trials are available in this field. Consequently, several treatment comparison nodes contain a limited number of included studies, and head-to-head RCTs are lacking for certain pairwise intervention comparisons. This may render SUCRA-based ranking results unstable, carrying potential risks of small-sample effects and publication bias. Meanwhile, the overall sample size incorporated in our analysis was insufficient, which may serve as one plausible explanation for the absence of statistically significant differences in long-term outcomes. Furthermore, inadequate sample size meant that individual subgroups could not fully encompass all five intervention modalities, namely MT, BDA, DCB, BMS and DES. As a result, network evidence loops could not be fully closed for each subgroup, and subgroup analyses were therefore not performed in the current study. Another important limitation relates to two included RCTs (19, 22), which enrolled mixed participants including patients with extracranial vertebral artery stenosis and thus deviated from our prespecified eligibility restricted to symptomatic intracranial atherosclerotic stenosis. Stratified outcome events according to lesion location were not reported in these original publications; hence we could not extract effect estimates limited only to intracranial stenosis patients. Nevertheless, our sensitivity analysis excluding these two trials (Table 4) showed that our main findings were not materially altered by this potential confounding bias.

Finally, the individual studies incorporated into the present meta-analysis also carry their own inherent limitations. For instance, the landmark SAMMPRIS trial had several notable drawbacks: a double-blind design could not be implemented; the intensive medical regimen adopted in the trial was highly stringent and could hardly be fully replicated within real-world clinical settings; and trial outcomes may have been confounded by unstable plaque status among participants at the time of endovascular intervention. Accordingly, further publication of high-quality randomized controlled trials is warranted to counterbalance the influence of these methodologically flawed studies.

## 5 Conclusion

This study demonstrated that among sICAS patients presenting with TIA and non-disabling stroke within 12 months, medical treatment exhibited significant superiority over BMS in terms of safety endpoints (stroke or death within 30 days), whereas no statistically significant differences were observed across all intervention strategies with regard to long-term outcomes (adverse events during follow-up). Although BDA and DES yielded favorable exploratory SUCRA probability rankings for long-term outcomes, these rankings do not establish clinical superiority and must be interpreted cautiously given that between-treatment differences were not statistically significant. Of note, Asian participants constituted the large majority of the pooled study population. Given well-documented ethnic differences in pathological features of intracranial atherosclerosis and treatment responses, caution is warranted when extrapolating these findings to non-Asian populations. Meanwhile, data concerning restenosis for drug-coated balloons and drug-eluting stents remain scarce, which underscores that additional randomized controlled trials (RCTs) are warranted to conduct risk–benefit assessments. We noted that numerous RCT protocols have currently been published. Moreover, head-to-head RCTs comparing DCB with medical treatment can be identified in the ClinicalTrials.gov database (32–34). This points to potential further advances within this research field.

## Data Availability

The original contributions presented in the study are included in the article/Supplementary material, further inquiries can be directed to the corresponding author.
