# Supplementary material for: Comparison of angioplasty, stents and conventional medical therapy for symptomatic intracranial atherosclerosis: a network meta-analysis
Source: Front Neurol. 2026 Sep 30;17:1848611. doi: 10.3389/fneur.2026.1848611 (PMC13661453; doi:10.3389/fneur.2026.1848611)
Supplement: Supplementary file 1 [file Table_1.DOCX]

Supplementary Material

**Supplementary Table 1.** Search strategy of cochrane central register of controlled trials (CENTRAL).

| # | Search | Results |
| --- | --- | --- |
| #1 | MeSH descriptor: [Intracranial Arteriosclerosis] explode all trees | 652 |
| #2 | (Arterioscleroses, Cerebral):ti,ab,kw OR (Arterioscleroses, Intracranial):ti,ab,kw OR (Arteriosclerosis, Cerebral):ti,ab,kw OR (Arteriosclerosis, Intracranial):ti,ab,kw OR (Atheroscleroses, Cerebral):ti,ab,kw OR (Atheroscleroses, Intracranial):ti,ab,kw OR (Atherosclerosis, Cerebral):ti,ab,kw OR (Atherosclerosis, Intracranial):ti,ab,kw OR (Cerebral Arterioscleroses):ti,ab,kw OR (Cerebral Arteriosclerosis):ti,ab,kw OR (Cerebral Atheroscleroses):ti,ab,kw OR (Cerebral Atherosclerosis):ti,ab,kw OR (Intracranial Arterioscleroses):ti,ab,kw OR (Intracranial Atheroscleroses):ti,ab,kw OR (Intracranial Atherosclerosis):ti,ab,kw | 997 |
| #3 | #1 OR #2 | 1377 |
| #4 | MeSH descriptor: [Drug-Eluting Stents] explode all trees | 2363 |
| #5 | MeSH descriptor: [Drug Therapy] explode all trees | 185728 |
| #6 | MeSH descriptor: [Angioplasty, Balloon] explode all trees | 5416 |
| #7 | MeSH descriptor: [Stents] explode all trees | 6670 |
| #8 | MeSH descriptor: [Self Expandable Metallic Stents] explode all trees | 95 |
| #9 | MeSH descriptor: [Angioplasty] explode all trees | 5990 |
| #10 | #4 OR #5 OR #6 OR #7 OR #8 OR #9 | 194146 |
| #11 | (Chemotherapies):ti,ab,kw OR (Chemotherapy):ti,ab,kw OR (coated balloon，drug):ti,ab,kw OR (DCB):ti,ab,kw OR (Drug Coated Stent):ti,ab,kw OR (Drug Coated Stents):ti,ab,kw OR (Drug Eluting Stent):ti,ab,kw OR (Drug Eluting Stents):ti,ab,kw OR (Drug Therapies):ti,ab,kw OR (drug therapy):ti,ab,kw OR (drug-coated balloons):ti,ab,kw OR (Drug-Coated Stent):ti,ab,kw OR (Drug-Coated Stents):ti,ab,kw OR (drug-eluting balloon):ti,ab,kw OR (Drug-Eluting Stent):ti,ab,kw OR (paclitaxel-coated balloon):ti,ab,kw OR (Pharmacotherapies):ti,ab,kw OR (Pharmacotherapy):ti,ab,kw OR (Stent, Drug-Coated):ti,ab,kw OR (Stent, Drug-Eluting):ti,ab,kw OR (Stents, Drug Coated):ti,ab,kw OR (Stents, Drug Eluting):ti,ab,kw OR (Stents, Drug-Coated):ti,ab,kw OR (Stents, Drug-Eluting):ti,ab,kw OR (Therapies, Drug):ti,ab,kw OR (Therapy, Drug):ti,ab,kw OR (Balloon Angioplasty):ti,ab,kw OR (Angioplasty, Balloon):ti,ab,kw OR (Dilation, Transluminal Arterial):ti,ab,kw OR (Arterial Dilations, Transluminal):ti,ab,kw OR (Arterial Dilation, Transluminal):ti,ab,kw OR (Dilations, Transluminal Arterial):ti,ab,kw OR (Transluminal Arterial Dilation):ti,ab,kw OR (Transluminal Arterial Dilations):ti,ab,kw OR (Stent):ti,ab,kw OR (Stents):ti,ab,kw OR (Self Expandable Metal Stents):ti,ab,kw OR (Self Expandable Metallic Stent):ti,ab,kw OR (Self Expandable Metal Stent):ti,ab,kw OR (Self Expandable Metallic Stents):ti,ab,kw OR (Angioplasty):ti,ab,kw OR (Angioplasties):ti,ab,kw OR (Endoluminal Repair):ti,ab,kw OR (Endoluminal Repairs):ti,ab,kw OR (Repair, Endoluminal):ti,ab,kw OR (Repairs, Endoluminal):ti,ab,kw OR (Angioplasty, Transluminal):ti,ab,kw OR (Transluminal Angioplasty):ti,ab,kw OR (Percutaneous Transluminal Angioplasty):ti,ab,kw OR (Angioplasty, Percutaneous Transluminal):ti,ab,kw OR (Transluminal Angioplasty, Percutaneous):ti,ab,kw | 643946 |
| #12 | #10 OR #11 | 687752 |
| #13 | MeSH descriptor: [Randomized Controlled Trial] explode all trees | 34 |
| #14 | (Randomized Controlled Trial):ti,ab,kw OR (Random):ti,ab,kw | 896046 |
| #15 | #13 OR #14 | 896046 |
| #16 | #15 AND #12 AND #3 | 353 |

**Supplementary Table 2.** Search strategy of Web of Science: Science citation index expanded.

| # | Search | Results |
| --- | --- | --- |
| #1 | Arterioscleroses, Intracranial (Topic) OR Arteriosclerosis, Intracranial (Topic) OR Intracranial Arterioscleroses (Topic) OR Cerebral Arteriosclerosis (Topic) OR Arterioscleroses, Cerebral (Topic) OR Arteriosclerosis, Cerebral (Topic) OR Cerebral Arterioscleroses (Topic) OR Cerebral Atherosclerosis (Topic) OR Atheroscleroses, Cerebral (Topic) OR Atherosclerosis, Cerebral (Topic) OR Cerebral Atheroscleroses (Topic) OR Intracranial Atherosclerosis (Topic) OR Atheroscleroses, Intracranial (Topic) OR Atherosclerosis, Intracranial (Topic) OR Intracranial Atheroscleroses (Topic) | 8694 |
| #2 | Randomized Controlled Trial (Topic) OR Random (Topic) | 1313458 |
|  |  |  |
| #3 | Drug Eluting Stents (Topic) OR Drug-Eluting Stent (Topic) OR Drug Eluting Stent (Topic) OR Stent, Drug-Eluting (Topic) OR Stents, Drug-Eluting (Topic) OR Stents, Drug Eluting (Topic) OR Drug-Coated Stents (Topic) OR Drug Coated Stents (Topic) OR Drug-Coated Stent (Topic) OR Drug Coated Stent (Topic) OR Stent, Drug-Coated (Topic) OR Stents, Drug-Coated (Topic) OR Stents, Drug Coated (Topic) OR Chemotherapy (Topic) OR drug therapy (Topic) OR Chemotherapies (Topic) OR Pharmacotherapy (Topic) OR Pharmacotherapies (Topic) OR Therapy, Drug (Topic) OR Drug Therapies (Topic) OR Therapies, Drug (Topic) OR drug-coated balloons (Topic) OR drug-eluting balloon (Topic) OR DCB (Topic) OR coated balloon，drug (Topic) OR paclitaxel-coated balloon (Topic) OR Balloon Angioplasty (Topic) OR Angioplasty, Balloon (Topic) OR Dilation, Transluminal Arterial (Topic) OR Arterial Dilations, Transluminal (Topic) OR Arterial Dilation, Transluminal (Topic) OR Dilations, Transluminal Arterial (Topic) OR Transluminal Arterial Dilation (Topic) OR Transluminal Arterial Dilations (Topic) OR Stent (Topic) OR Stents (Topic) OR Self Expandable Metal Stents (Topic) OR Self Expandable Metallic Stent (Topic) OR Self Expandable Metal Stent (Topic) OR Self Expandable Metallic Stents (Topic) OR Angioplasty (Topic) OR Angioplasties (Topic) OR Endoluminal Repair (Topic) OR Endoluminal Repairs (Topic) OR Repair, Endoluminal (Topic) OR Repairs, Endoluminal (Topic) OR Angioplasty, Transluminal (Topic) OR Transluminal Angioplasty (Topic) OR Percutaneous Transluminal Angioplasty (Topic) OR Angioplasty, Percutaneous Transluminal (Topic) OR Transluminal Angioplasty, Percutaneous (Topic) | 1178973 |
| #4 | #1 AND #2 AND #3 | 88 |
| #5 | #1 AND #2 AND #3 | 88 |

**Supplementary Table 3.** Search strategy of PubMed.

**Supplementary Table 4.** Search strategy of Embase.

| # | Search | Results |
| --- | --- | --- |
| #1 | 'cerebral atherosclerosis'/exp | 8866 |
| #2 | 'arterioscleroses, cerebral':ab,ti OR 'arterioscleroses, intracranial':ab,ti OR 'arteriosclerosis, cerebral':ab,ti OR 'arteriosclerosis, intracranial':ab,ti OR 'atheroscleroses, cerebral':ab,ti OR 'atheroscleroses, intracranial':ab,ti OR 'atherosclerosis, cerebral':ab,ti OR 'atherosclerosis, intracranial':ab,ti OR 'cerebral arterioscleroses':ab,ti OR 'cerebral arteriosclerosis':ab,ti OR 'cerebral atheroscleroses':ab,ti OR 'cerebral atherosclerosis':ab,ti OR 'intracranial arterioscleroses':ab,ti OR 'intracranial atheroscleroses':ab,ti OR 'intracranial atherosclerosis':ab,ti | 3130 |
| #3 | #1 OR #2 | 10103 |
| #4 | 'randomized controlled trial'/exp | 1140527 |
| #5 | 'randomized controlled trial':ab,ti OR 'random':ab,ti | 750106 |
| #6 | #4 OR #5 | 1674017 |
| #7 | 'drug eluting stent'/exp OR 'drug therapy'/exp OR 'percutaneous transluminal angioplasty'/exp OR 'stent'/exp OR 'angioplasty'/exp | 4886356 |
| #8 | 'drug eluting stents':ab,ti OR 'drug-eluting stent':ab,ti OR 'drug eluting stent':ab,ti OR 'stent, drug-eluting':ab,ti OR 'stents, drug-eluting':ab,ti OR 'stents, drug eluting':ab,ti OR 'drug-coated stents':ab,ti OR 'drug coated stents':ab,ti OR 'drug-coated stent':ab,ti OR 'drug coated stent':ab,ti OR 'stent, drug-coated':ab,ti OR 'stents, drug-coated':ab,ti OR 'stents, drug coated':ab,ti OR 'chemotherapy':ab,ti OR 'drug therapy':ab,ti OR 'chemotherapies':ab,ti OR 'pharmacotherapy':ab,ti OR 'pharmacotherapies':ab,ti OR 'therapy, drug':ab,ti OR 'drug therapies':ab,ti OR 'therapies, drug':ab,ti OR 'drug-coated balloons':ab,ti OR 'drug-eluting balloon':ab,ti OR 'dcb':ab,ti OR 'coated balloon，drug':ab,ti OR 'paclitaxel-coated balloon':ab,ti OR 'balloon angioplasty':ab,ti OR 'angioplasty, balloon':ab,ti OR 'dilation, transluminal arterial':ab,ti OR 'arterial dilations, transluminal':ab,ti OR 'arterial dilation, transluminal':ab,ti OR 'dilations, transluminal arterial':ab,ti OR 'transluminal arterial dilation':ab,ti OR 'transluminal arterial dilations':ab,ti OR 'stent':ab,ti OR 'stents':ab,ti OR 'self expandable metal stents':ab,ti OR 'self expandable metallic stent':ab,ti OR 'self expandable metal stent':ab,ti OR 'self expandable metallic stents':ab,ti OR 'angioplasty':ab,ti OR 'angioplasties':ab,ti OR 'endoluminal repair':ab,ti OR 'endoluminal repairs':ab,ti OR 'repair, endoluminal':ab,ti OR 'repairs, endoluminal':ab,ti OR 'angioplasty, transluminal':ab,ti OR 'transluminal angioplasty':ab,ti OR 'percutaneous transluminal angioplasty':ab,ti OR 'angioplasty, percutaneous transluminal':ab,ti OR 'transluminal angioplasty, percutaneous':ab,ti | 1257529 |
| #9 | #7 OR #8 | 5236596 |
| #10 | #3 AND #6 AND #9 | 171 |

**Supplementary Table 5.** Search strategy of CBM.

| # | Search | Results |
| --- | --- | --- |
| #1 | "Intracranial atherosclerosis" [Common field: Smart] OR "Cerebral atherosclerosis" [Common field: Smart] OR "Intracranial atherosclerosis" [Common field: Smart] | 15766 |
| #2 | "Intracranial atherosclerosis" [Unweighted: Extended] | 14032 |
| #3 | (#2) OR (#1) | 15766 |
| #4 | "Randomized controlled trials" [Unweighted: Extended] | 199630 |
| #5 | "Randomized controlled trials" [Common field: Smart]  OR "Randomized" [Common field: Smart] OR "RCT"[Common field: Smart] | 2123145 |
| #6 | (#5) OR (#4) | 2123145 |
| #7 | ((((("Drug-eluting stents"[Unweighted: Extended]) OR "Pharmacotherapy"[Unweighted: Extended]) OR "Balloon angioplasty"[Unweighted: Extended]) OR "Angioplasty"[Unweighted: Extended]) OR "Stents"[Unweighted: Extended]) | 586957 |
| #8 | ("Transluminal angioplasty"[Common field: Smart] OR "Percutaneous transluminal angioplasty"[Common field: Smart] OR "Vascular revascularization"[Common field: Smart] OR "Stents"[Common field: Smart] OR "Drug-eluting stents"[Common field: Smart] OR "Drug-coated balloon"[Common field: Smart] OR "DCB"[Common field: Smart] OR ("Drug-eluting stents"[Common field: Smart] OR "Chemotherapy"[Common field: Smart] OR "Pharmacotherapy"[Common field: Smart] OR "Balloon angioplasty"[Common field: Smart] OR "Transluminal arterial dilatation"[Common field: Smart]) | 1051147 |
| #9 | (#8) OR (#7) | 1051147 |
| #10 | (#9) AND (#6) AND (#3) | 503 |

**Supplementary Table 6.** Search strategy of CNKI.

| # | Search | Results |
| --- | --- | --- |
| #1 | (SU %= 'Intracranial atherosclerosis' OR SU %= 'Cerebral atherosclerosis' OR SU %= 'Cerebral atherosclerosis' OR) AND (SU %= 'Drug-eluting stents' OR SU %= 'Chemotherapy' OR SU %= 'Pharmacotherapy' OR SU %= 'DCB' OR SU %= 'Drug-coated balloon' OR SU %= 'Drug-eluting stents' OR SU %= 'Drug-coated balloon' OR SU %= 'Balloon angioplasty' OR SU %= 'Stents' OR SU %= 'Transluminal angioplasty' OR SU %= 'Percutaneous transluminal angioplasty' OR SU %= 'Vascular revascularization' OR SU %= 'Transluminal arterial dilatation' OR SU %= 'Angioplasty') AND (AB = 'Randomized controlled trials' OR AB = 'Randomized' OR AB = 'RCT') | 233 |

**Supplementary Table 7.** Search strategy of VIP.

| # | Search | Results |
| --- | --- | --- |
| #1 | M=(Intracranial atherosclerosis + Cerebral atherosclerosis + Cerebral atherosclerosis) * R=(Drug-eluting stents + Chemotherapy + Pharmacotherapy + Drug-coated balloon + Balloon angioplasty + Transluminal arterial dilatation + Transluminal angioplasty + Percutaneous transluminal angioplasty + Vascular revascularization + Angioplasty + DCB + Drug-coated balloon + Stents) * R=(Randomized controlled trials + Randomized + RCT) | 174 |

**Supplementary Table 8.** Search strategy of Wanfang.

| # | Search | Results |
| --- | --- | --- |
| #1 | Topic: ((("Intracranial atherosclerosis" or "Cerebral atherosclerosis") and ("Drug-eluting stents" or "Chemotherapy" or "Pharmacotherapy" or "Drug-coated balloon" or "Balloon angioplasty" or "DCB" or "Transluminal arterial dilatation" or "Balloon angioplasty" or "Transluminal angioplasty" or "Percutaneous transluminal angioplasty" or "Vascular revascularization" or "Stents") and ("Randomized controlled trials" or "Randomized" or "RCT")) | 69 |

**Supplementary Table 9.** Study level risk of bias assessment using Cochrane risk of bias tool 2.0 for assessing risk of bias of randomized clinical trials.

| **Unique ID** | **Reviewer** | **Study ID** | **Weight** | **Randomization process** | **Deviations from intended interventions** | **Mising outcome data** | **Measurement of the outcome** | **Selection of the reported result** | **Overall Bias** |
| --- | --- | --- | --- | --- | --- | --- | --- | --- | --- |
| Sun 2024 | Wang | NCT03703635 | 1 | Low | Low | Low | Low | Low | Low |
| Ma 2025 | Wang | NCT04631055 | 1 | Low | Low | Low | Low | Low | Low |
| Jia 2022 | Wang | NCT02578069 | 1 | Low | Low | Low | Low | Low | Low |
| Gao 2022 | Wang | NCT01763320 | 1 | Low | Low | Low | Low | Low | Low |
| Bonaventure 2025 | Wang | CUHK-CCT00116 | 1 | Low | Low | Low | Low | Low | Low |
| Marc I 2011 | Wang | NCT00576693 | 1 | Low | Low | Low | Low | Low | Low |
| Xiao Wen 2014 | Wang | NA | 1 | Some concerns | Low | Low | Low | Low | Some concerns |
| Huang Xuxin 2022 | Wang | NA | 1 | Some concerns | Low | Low | Low | Low | Some concerns |
| Zaidat 2015 | Wang | NCT00816166 | 1 | Low | Low | High | Some concerns | Low | High |
| Si 2022 | Wang | ChiCTR2200055925 | 1 | Low | Low | High | Low | Low | High |
| He 2019 | Wang | ChiCTR-IIR-16009115 | 1 | High | Low | Low | Low | Low | High |

**Supplementary Table 10.** Safety outcomes

| BDA | 0.84 (0.14,5.04) | 1.14 (0.26,4.94) | 0.49 (0.15,1.62) | 1.04 (0.30,3.68) |
| --- | --- | --- | --- | --- |
| 1.20 (0.20,7.21) | DCB | 1.37 (0.31,6.03) | 0.59 (0.15,2.27) | 1.25 (0.35,4.50) |
| 0.87 (0.20,3.78) | 0.73 (0.17,3.22) | DES | 0.43 (0.18,1.02) | 0.91 (0.43,1.93) |
| 2.02 (0.62,6.64) | 1.69 (0.44,6.51) | 2.31 (0.98,5.44) | MT | 2.11 (1.40,3.20) |
| 0.96 (0.27,3.37) | 0.80 (0.22,2.88) | 1.09 (0.52,2.31) | 0.47 (0.31,0.72) | BMS |

**Supplementary Table 11.** Long-term outcomes

| BDA | 1.15 (0.13,10.46) | 2.84 (0.60,13.40) | 2.99 (0.52,17.37) |
| --- | --- | --- | --- |
| 0.87 (0.10,7.95) | DES | 2.48 (0.51,11.97) | 2.61 (0.68,9.98) |
| 0.35 (0.07,1.66) | 0.40 (0.08,1.95) | MT | 1.05 (0.46,2.41) |
| 0.33 (0.06,1.94) | 0.38 (0.10,1.46) | 0.95 (0.41,2.17) | BMS |

**Supplementary Table 12.** Safety outcomes **CINeMA_**

| Comparison | Number of studies | Within-study bias | Reporting bias | Indirectness | Imprecision | Heterogeneity | Incoherence | Confidence rating | Reason(s) for downgrading |
| --- | --- | --- | --- | --- | --- | --- | --- | --- | --- |
| BDA:MT | 1 | No concerns | Low risk | No concerns | Major concerns | No concerns | Major concerns | Moderate | Downgraded for imprecision (wide confidence intervals) and network incoherence. |
| BMS:DCB | 1 | No concerns | Low risk | No concerns | Major concerns | No concerns | Major concerns | Moderate | Downgraded for imprecision (wide confidence intervals) and network incoherence. |
| BMS:DES | 3 | Some concerns | Low risk | No concerns | Major concerns | No concerns | Major concerns | Low | Downgraded for some concerns for within‑study bias, imprecision (wide confidence intervals) and network incoherence. |
| BMS:MT | 5 | No concerns | Low risk | No concerns | No concerns | No concerns | Major concerns | Moderate | Downgraded for network incoherence. |
| BDA:BMS | 0 | No concerns | Low risk | No concerns | Major concerns | No concerns | Major concerns | Moderate | Downgraded for imprecision (wide prediction/confidence intervals) and network incoherence; comparison relies entirely on indirect evidence. |
| BDA:DCB | 0 | No concerns | Low risk | No concerns | Major concerns | No concerns | Major concerns | Moderate | Downgraded for imprecision (wide prediction/confidence intervals) and network incoherence; comparison relies entirely on indirect evidence. |
| BDA:DES | 0 | No concerns | Low risk | No concerns | Major concerns | No concerns | Major concerns | Moderate | Downgraded for imprecision (wide prediction/confidence intervals) and network incoherence; comparison relies entirely on indirect evidence. |
| DCB:DES | 0 | No concerns | Low risk | No concerns | Major concerns | No concerns | Major concerns | Moderate | Downgraded for imprecision (wide prediction/confidence intervals) and network incoherence; comparison relies entirely on indirect evidence. |
| DCB:MT | 0 | No concerns | Low risk | No concerns | Major concerns | No concerns | Major concerns | Moderate | Downgraded for imprecision (wide prediction/confidence intervals) and network incoherence; comparison relies entirely on indirect evidence. |
| DES:MT | 0 | Some concerns | Low risk | No concerns | Major concerns | No concerns | Major concerns | Low | Downgraded for some concerns for within‑study bias, imprecision (wide prediction/confidence intervals) and network incoherence; comparison relies entirely on indirect evidence. |

**Supplementary Table 13.** Long-term outcomes CINeMA

| Comparison | Number of studies | Within-study bias | Reporting bias | Indirectness | Imprecision | Heterogeneity | Incoherence | Confidence rating | Reason(s) for downgrading |
| --- | --- | --- | --- | --- | --- | --- | --- | --- | --- |
| BDA:MT | 1 | No concerns | Low risk | No concerns | No concerns | Major concerns | Major concerns | Low | Downgraded for substantial between‑study heterogeneity and network incoherence. |
| BMS:DES | 2 | Some concerns | Low risk | No concerns | No concerns | Major concerns | Major concerns | Low | Downgraded for some concerns for within‑study bias, substantial between‑study heterogeneity and network incoherence. |
| BMS:MT | 4 | Some concerns | Low risk | No concerns | Major concerns | No concerns | Major concerns | Low | Downgraded for some concerns for within‑study bias, imprecision (wide confidence intervals) and network incoherence. |
| BDA:BMS | 0 | No concerns | Low risk | No concerns | No concerns | Major concerns | Major concerns | Low | Downgraded for substantial between‑study heterogeneity and network incoherence; comparison relies entirely on indirect evidence. |
| BDA:DES | 0 | No concerns | Low risk | No concerns | Major concerns | No concerns | Major concerns | Low | Downgraded for imprecision (wide prediction/confidence intervals) and network incoherence; comparison relies entirely on indirect evidence. |
| DES:MT | 0 | Some concerns | Low risk | No concerns | Major concerns | No concerns | Major concerns | Low | Downgraded for some concerns for within‑study bias, imprecision (wide prediction/confidence intervals) and network incoherence; comparison relies entirely on indirect evidence. |

**Supplementary Table 14.** Outcomes and assessment time point of each study

| Study | Safety outcomes | Efficacy outcome and assessment time point |
| --- | --- | --- |
| Sun 2024 | Any stroke or all-cause death within 30 days after enrollment | Any ischemic stroke of the qualifying artery within 1 year after enrollment |
|  |  |  |
| Ma 2025 | Post-procedure target vessel-related stroke (hemorrhage or ischemia) or death at 30 days | NA |
|  |  |  |
| Jia 2022 | Any stroke or death within 30 days after the procedure | Ischemic stroke in target vessel territory within 1 year after enrollment |
|  |  |  |
| Gao 2022 | Stroke or death within 30 days after enrollment | Stroke in territory of qualifying artery within 1 year |
|  |  |  |
| Ip 2025 | Composite of any stroke or death within 30 days | Ischemic stroke in the same territory within 1 year |
|  |  |  |
| Chimowitz 2011 | Stroke or death within 30 days after enrolment | NA |
|  |  |  |
| Xiao 2014 | Stroke or death within 30 days after enrollment | NA |
|  |  |  |
| Huang 2022 | NA | Ischemic stroke in the same territory within 1 year |
|  |  |  |
| Zaidat 2015 | Stroke or death within 30 days after enrollment | Stroke in the same territory within 1 year of randomization |
|  |  |  |
| Si 2022 | Stroke in any territory and death within 30 days of randomization | Ischemic stroke and TIA in the same territory as the presenting event (distal to the target lesion) within 1 year of randomization |
|  |  |  |
| He 2019 | Surgical complications within 30 days after procedure | Stroke ipsilateral to the target vertebral artery cerebrovascular and cardiovascular events, and serious adverse events within 12 months after operation |
|  |  |  |

**Supplementary Figure 1.** Cumulative ranking curve plots of different treatment for Safety Outcomes in range network.

**Supplementary Figure 2.** Cumulative ranking curve plots of different treatment for Long-term Outcomes in range network.

**Supplementary Figure 3.** Risk of bias contribution by intervention group in Safety Outcomes.

**Supplementary Figure 4.** Overall risk of bias by treatment comparison in Safety Outcomes.

**Supplementary Figure 5.** Risk of bias contribution by intervention group in Long-term Outcomes.

**Supplementary Figure 6.** Overall risk of bias by treatment comparison in Long-term Outcomes.
